# Treatment for sickle cell disease in Africa: should we invest in haematopoietic stem cell transplantation?

**DOI:** 10.11604/pamj.2014.18.46.3923

**Published:** 2014-05-13

**Authors:** Gift Pule, Ambroise Wonkam

**Affiliations:** 1 Division of Human Genetics, Department of Clinical Laboratory Sciences, Faculty of Health Sciences, University of Cape Town (UCT), Cape Town, South Africa

**Keywords:** Sickle Cell Disease, hydroxyurea, haematopoietic stem cells transplantation, Africa

## Commentary

### Epidemiology and burden of SCD

Sickle cell disease is a life-long genetic disease that begins in childhood, affecting the structure of erythrocytes, altering the healthy biconcave shape to a crescent shape, leading to the blockage of veins, thereby resulting in organ damage [1]. There is strong correlation between the frequency of the HbS gene and the historical distribution and incidences of malaria due to the partial HbS-carrier resistance to Plasmodium falciparum malaria [2]. Indeed, Sickle Cell Anaemia mutation (HbS gene) appears to have occurred independently in 4 regions in Africa, defined by four haplotypes (Senegal, Benin, Bantu and Cameroon haplotypes)[3]. SCD is prevalent among indigenous populations in tropical regions of Africa and Asia; 305800 births with SCD are estimated to occur annually, nearly 67% of which take place in Africa. Sickle Cell Anaemia (SCA; the homozygousHbSS state) is by far the most prevalent and severe form of SCD [4]. Many countries in Africa have developed a national control program for SCD, however provisions of neonatal screening are rare [5] and development of specialized centres for lifelong medical care and surveillance have yet to become part of many SCD health systems, and in the absence of universal medical insurance coverage in many African countries, the chronic care of SCD patients is therefore dependent on financial support and care-giving by family member [6]. In addition, vaso-occlusive painful events, silent and overt stroke that occur in SCD could potentially contribute to functional limitations and poor academic achievement of affected children. Indeed, it was reported in Cameroon that up to 37.5% of participants’ SCD-affected children had mild-to-severe cognitive deficits, and there was a significant effect on executive functions and attention [7]. Poor health status of children with SCD could also reduce caregivers’ employability and worsen the socioeconomic burden on families. Indeed up to 24.3% of caregivers in the USA missed two or more days of work per 3 days-hospital admission of their children [8], and the morbidity of a painful event continued after discharge from hospital [8, 9]. Similar findings were also recently reported in Cameroon[6].

The mortality rate associated with SCD has remained high in Africa, despite the use of appropriate interventions to manage the various forms of crises [10]. In the USA and Europe, who together account for less than 8% of the global disease burden of SCD, new-born screening, pneumococcal immunization, prophylactic penicillin and most importantly HU treatment, have decreased morbidity and mortality and thus increasing survival rates from childhood diagnoses to over 95% [4, 11]. In stark contrast, as of 2010, sub-Saharan Africa accounted for 75.5% of the global number of new-borns with SCD, where most of these children die before age 5 due to a myriad of socio-economic factors and a poor public healthcare system [4]. The limited early detection and treatment initiatives that have been implemented in Africa result in high death rates before the age of 5 [10, 12]. These statistics highlight the imperative necessity of research and translational medicine in to improve the burden through better care and potentially a cure of SCD in Africa.

### Treatment approaches

There are five treatment approaches for SCD that are tailored to the clinical phenotype of a patient, namely supportive, symptomatic, preventative, abortive and curative approaches [13]. The supportive approach is the most common, aimed at the management of the patient and such an approach includes a balanced diet, hydration and folic acid supplementation. Blood transfusions, analgesia and antibiotics are typed as symptomatic approaches because their function is to alleviate specific SCD symptoms. The preventative approach is taken to preclude the occurrence of disease complications such as pneumonia and influenza vaccination, hydroxyurea for the induction of foetal haemoglobin (HbF) and blood transfusions to avert primary and secondary stroke episodes [14]. Nitric oxide (NO) is the only accepted agent for the abortive approach, reported to completely terminate of chronic pain episodes in some SCD patients [15]. Lastly, the curative approach is the ultimate goal for all genetic disorders, intended to correct the disease-causing mutation and prevent all complications. Currently, transplantation of haematopoietic stem cells (HSCs) is the only accepted curative treatment for SCD. Below, we briefly describe the 3 current major strategies for effective treatment of SCD, namely blood transfusion, hydroxyurea (HU) and HSC transplantation.

*Blood transfusion*: Blood transfusions improve the oxygen-carrying capacity and oxygen-delivery efficiency of blood to the tissues and decrease the blood concentration of sickle cells and improve perfusion of tissue microvasculature. Transfusions are typically used to ameliorate chronic anaemia and pain episodes [16], and are highly effective in patients with sporadic episodes of severe anaemia by preventing organ damage. Although transfusion can be applied as a preventative, abortive or curative approach for treatment of certain complications, they have many drawbacks. There are few blood transfusion services across the continent with the capacity to cope with the demand for regular transfusions [17, 18]. Furthermore, widespread diseases such as HIV/AIDS and TB reduce the number of possible donors as well as the limited technical and financial support offered by the state's department of health. Although not specific to Africa, graft versus host disease (GVHD), even after alloimmunization [19], is one of the challenges surrounding transfusions. Other possible complications include transfusion-induced haemolytic reaction or autoimmune hyper-haemolysis [20] and blood volume and iron overload [21]. This evidence suggests that although useful and effective in certain circumstances, blood transfusion alone is not a sufficient treatment for SCD nor is it curative. Therefore below, we discuss the use hydroxyurea as a drug treatment for SCD.

*Hydroxyurea*: Hydroxyurea is an oral, cytotoxic, anti-metabolic and -neoplastic drug for principal haematological disorders. The first clinical application of HU was in 1984 [22] and since then have been supported by numerous clinical trials demonstrating clinical efficacy and increase in survival rates and life expectancy [23], protection against cerebrovascular disease [24], long term drug safety, capacity to prevent organ damage, reduce morbidity and mortality in school-age children [25], toddlers [26] and infants [27]. The most notable evidence for the clinical efficacy of HU came from the Multicentre Study of Hydroxyurea (MSH) clinical trial [28]. The trial showed decreases in the frequency of painful episodes, acute chest syndrome, hospitalization and transfusions. Following FDA approval in 1998, 2007 saw HU receive approval from the European Medicines Agency (EMeA) as treatment for both adults and children with SCD. One year later, the National Institutes of Health Office of Medical Applications of Research (NIH-OMAR) and the Agency of Healthcare Research and Quality (AHRQ) both declared HU as an effective drug treatment for adults and children with SCD [29]. However, despite these results and the National Institutes of Health (NIH) recommendation for the use of HU in adults and children with SCD [30], HU is still underutilized [31]. This was confirmed by the NIH Consensus Development Conference statement [29] leading to studies investigating the barriers to the widespread use of HU. In surveys of the American Society of Paediatric Haematology and Oncologyand Florida and North Carolina's Haematologists and Oncologists, it was reported that the most common barrier to the prescription of HU for SCD is compliance from the patients and their families [32, 33]. Similar barriers to the use of HU in children have also been reported [34] with the age of the children being one of the major barriers to prescription, despite data from the Hydroxyurea to Prevent Organ Damage in Children with SCD confirming the safety of HU in young children [35]. Similarly, HU is still not included within national guidelines for use in children below age 5 in some West African countries such as Kenya where the disease burden is highest [36, 37], and recent report suggest that less than 5% of SCD patients in Cameroon ever used HU [6]. Furthermore, a recent review on the efficacy of HU in preventing SCD complications revealed that the majority of the studies were conducted in high-income countries with just 2 studies completed low-income countries, Tunisia and India [36]. This is illustrative of the apparent disproportion between regions of high disease burden and investment into medical research and intervention. Clinical trials investigating the effectiveness of HU in Africa are imperative as well as overcoming the barriers to the necessary utilization of HU in both children and adults with SCD and more importantly, further exploring curative modalities such as stem cell transplantation.

*Haematopoietic stem cell transplantation*: Currently, allogeneic HSC transplantation is the most successful curative treatment for SCD [38]. This method was first reported in 1984 with the HSCs derived from bone marrow, transplanted and successfully curing a patient with SCD and acute myelogenousleukemia[39, 40]. To date, approximately 250 individuals have received HSC transplantation for SCD worldwide [41]. Transplantations can be autologous or allogeneic. Autologous HSC transplantation is used depending on the severity of the presenting symptoms as this approach averts immune rejection. After comprehensive screening, the patient receives immunosuppressive and myeloblative treatment [41]. Although the myeloblative treatment has been associated with secondary toxicity-related complications such in infertility and graft versus host disease (GVHD) [41], immunosuppressants such as cyclosporine and methotrexate have been used to prevent GVHD [42]. Using the Worldwide Network for Blood and Marrow Transplantation and the World Health Organization (WHO), the total number of first-time HSC transplantation between 2006 and 2008 were reported by WHO regional offices [43]. The findings were indicative of a general increase in the number of HSC transplants worldwide, with the highest in Asia. Of all 146 808 HSC transplants between 2006 and 2008, Europe and the Americas accounted for 50.7 and 28.9% respectively, whereas both Africa and the eastern Mediterranean region accounted for 2.7%, most of which were conducted in the United Arab Emirates, Qatar and Egypt [43]. Using a linear regression, they showed strong correlations between rates of transplantation and government healthcare expenditure, gross national income per capita and overall infrastructure in the country [43]. As encouraging as the general hike in transplant rates is, the above correlations bid developing countries, particularly in Africa, more challenges to meet the extraordinary needs for HSC transplants. In contrast, Egypt has since 1989 initiated a Stem Cell Transplant (SCT) program for all haematological disorders, which by 2007 had performed 1362 transplants, 80% of which were allogeneic [44]. The average 25 - 30% sibling HLA match is generally higher (about 40%) owing to the typical larger family size in most African communities. Egypt has 8 transplant centres performing about 210 transplants annually, the biggest being the Nasser Institute, which has completely shifted from bone marrow to peripheral blood as a stem cell source, a seemingly better option in the developing world. β-thalassemia major, being the most common haemolytic anaemia in Egypt, saw compelling economic support for establishing a SCT program, which now has an overall and disease-free rates of 90 and 85%, respectively [44]. The SCT program in Egypt validates the feasibility of such programs in the third world and could be the focal point of a regional collaboration to initiate and develop this practice to cure SCD in Sub-Saharan African settings, and its successful transplant rates advocate for the establishment of similar programs across Africa.

### Advocacy for Haematopoietic stem cell transplantationcentres

Every 2 years, Africa sees the birth of a number of SCD-affected children equal to the sum of the American and European populations affected by SCD, where most of these children die before age 5 [45]. One of the major obstacles to the management of SCD, particularly in developing countries, is the reluctance of governments and international healthcare agencies to accept that SCD is a worldwide health problem, comparable to that of communicable diseases and other major global disease such as diabetes and hypertension [46]. The Nasser Institute in Egypt serves as a model from which we advocate for the establishment of similar transplantation centres across the continent. Although the average cost of SCT in Egypt is 15 000 USD, this cost is completely sponsored by the Ministry of Health or medical insurance [44] due to a collaborative commitment between government and their private sector. We strongly urge such commitments to developing research, science and technology in order to build the necessary scientific and research capacity in Africa. We advocate for a joint effort from Cape and Cairo, to initiate a centre for HSC transplantation in Africa, with the respective governments, members of the private sector and pharmaceutical industry, leading researchers and clinicians as stakeholders. We propose an intra-Africa collaboration and pooling of resources towards developing a transplantation centre that will be mandated with developing cost-effective procedures for HSC transplants for haematological disorders like SCD and β-thalassemia; large-scale clinical trials and follow-up studies, initiating epidemiological studies of haematological disorders in Africa, studies on health-related quality of life (HRQL) and patient survival rates, commercialization and distribution of treatments and therapies across the continent and most importantly, building technological and research capacity in Africa through teaching and training laboratory technicians and researchers. Such an initiative would also strengthen collaborative relationships across research and academic institutions in Africa, leading to a combined database of patients, information and experimental procedures, which will go towards standardizing practices within the field. The centre could initiate research towards developing disease models and investigating manifestation of diseases and accounting for previously undocumented African polymorphisms, develop an Afrocentric pharmacogenomics knowledge base including methods of drug design, predictive responder indexes and toxicity studies. This centre could also give rise to other initiatives such as umbilical cord blood and stem cell banks and continue building scientific and technological infrastructure in Africa. Furthermore, it will lead to improving blood transfusion services across the continent, incite further investigation of drug treatments like HU through clinical trials such as the NIH's Novel use of HU in an African Region with Malaria (NOHARM) and a better understanding of disease-modifying polymorphisms within various African populations.

## Conclusion

The data available on clinical trials and reports conducted outside of Africa is illustrative that a range of treatments are available and have been successful at curing SCD. What is required is the implementation of strategies to affordably avail these treatments in Africa, particularly HU and ultimately, HSC transplantation, through a collective effort from researchers, physicians, state departments of health and the private sector. This approach is likely to foster collaborative healthcare and research networks across the continent, ensuring the continued development of scientific and technological capacity in Africa to effectively manage the high disease burden of haematological disorders such as SCD.
